# Enhanced late-outgrowth circulating endothelial progenitor cell levels in rheumatoid arthritis and correlation with disease activity

**DOI:** 10.1186/ar2934

**Published:** 2010-02-16

**Authors:** Vanina Jodon de Villeroché, Jérome Avouac, Aurélie Ponceau, Barbara Ruiz, André Kahan, Catherine Boileau, Georges Uzan, Yannick Allanore

**Affiliations:** 1 INSERM U781, Paris Descartes University, Necker Hospital, 149 Rue de Sevres, 75015 Paris, France; 2 Rheumatology A department, Cochin Hospital, APHP, 27 rue du faubourg Saint Jacques, 75014 Paris, France; 3 UVSQ University, Biochemistry, Hormonology and Molecular Genetics Department, Ambroise Paré Hospital, AP-HP, 9 avenue Charles-de-Gaulle, 92100 Boulogne-Billancourt, France; 4 INSERM U972, Paul Brousse Hospital, 14 avenue Paul Vaillant-Couturier, BP200, 94804 Villejuif, France

## Abstract

**Introduction:**

Angiogenesis and vasculogenesis are critical in rheumatoid arthritis (RA) as they could be a key issue for chronic synovitis. Contradictory results have been published regarding circulating endothelial progenitor cells (EPCs) in RA. We herein investigated late outgrowth EPC sub-population using recent recommendations in patients with RA and healthy controls.

**Methods:**

EPCs, defined as Lin-/7AAD-/CD34+/CD133+/VEGFR-2+ cells, were quantified by flow cytometry in peripheral blood mononuclear cells (PBMCs) from 59 RA patients (mean age: 54 ± 15 years, disease duration: 16 ± 11 years) and 36 controls (mean age: 53 ± 19 years) free of cardiovascular events and of cardiovascular risk factors. Concomitantly, late outgrowth endothelial cell colonies derived from culture of PBMCs were analyzed by colony-forming units (CFUs).

**Results:**

RA patients displayed higher circulating EPC counts than controls (median 112 [27 to 588] vs. 60 [5 to 275]) per million Lin- mononuclear cells; *P* = 0.0007). The number of circulating EPCs positively correlated with disease activity reflected by DAS-28 score (r = 0.43; *P* = 0.0028) and lower counts were found in RA patients fulfilling remission criteria (*P* = 0.0069). Furthermore, late outgrowth CFU number was increased in RA patients compared to controls. In RA, there was no association between the number of EPCs and serum markers of inflammation or endothelial injury or synovitis.

**Conclusions:**

Our data, based on a well characterized definition of late outgrowth EPCs, demonstrate enhanced levels in RA and relationship with disease activity. This supports the contribution of vasculogenesis in the inflammatory articular process that occurs in RA by mobilization of EPCs.

## Introduction

Rheumatoid arthritis (RA) is a chronic and destructive inflammatory disease affecting the joints. RA is now well known to be associated with striking neovascularization developed in inflammatory joints [1]. Indeed, angiogenesis, leading to an increased number of synovial vessels through local endothelial cells, is a cornerstone of synovial hyperplasia occurring in RA. Disturbances in endothelial cell turnover and apoptosis as well as in angiogenic factors such as vascular endothelial growth factor (VEGF) have been reported in RA synovium [2,3]. However, despite the abundant synovial vasculature, there are areas of synovial hypoxia contributing to synovial and cartilage damage [4,5]. Hypoxia is highly suggested to activate the angiogenic cascade, thereby contributing to the perpetuation of RA synovitis [6].

In addition to angiogenesis issued from resident cells, cells derived from bone marrow and named circulating endothelial progenitor cells (EPCs) are able to promote new blood vessel formation (vasculogenesis) and may therefore contribute to RA synovitis [7].

EPCs were originally identified by (a) the expression of markers shared with hematopoietic stem cells such as CD34 and CD133, (b) specific endothelial cell markers such as KDR (vascular endothelial growth factor receptor-2 [VEGFR-2] or kinase-insert domain receptor), and (c) their capacity to differentiate into functional endothelial cells [8-10]. However, there is no consensus on the precise definition of EPCs [11]. Evidence showed that there is more than one endothelial progeny, monocytic versus hemangioblastic, within the circulating blood, and two distinct cell types of EPCs are currently recognized according to their growth characteristics and morphological appearance: early-outgrowth EPCs and late-outgrowth EPCs [7,12].

In the currently available human studies, variations in the level of circulating EPCs were reported in different diseases affecting the vascular system and were suggested to be a biomarker for vascular function and tumor progression [13,14]. In the field of RA, contradictory results have been reported. Indeed, some studies suggested a lower circulating EPC number in RA patients compared with controls [9,10], but conversely, some others reported higher values [15], and finally some other reports did not find any difference [8,16]. Several studies have shown an increase of EPCs within the RA synovial tissue [17,18].

Altogether, these observations underline the difficulty of accurately quantifying EPC populations. The major issue is the identification of the different types of circulating endothelial cells (CECs) issued respectively from the vessel wall or from bone marrow progenitors. The use of accurate methods allowing the detection of rare events by flow cytometry is thus critical. Within this context, our group contributed to recommendations aiming at the improvement of EPC detection and characterization [19]. In line with these latter recommendations and our background in systemic sclerosis [20], we focused on late-outgrowth EPCs that represent the progenitors with the more genuine endothelial properties. In parallel to EPCs, CECs detached from vessel walls (CECs) may also be a relevant biomarker of vascular disease. We hypothesized that coupled raised levels of these two populations may reflect the vascular status of the disease and thus represent innovative biomarkers. Therefore, our aims were (a) to enumerate EPCs and late-outgrowth endothelial colony formation in RA patients and controls, (b) to assess correlations between EPC counts, CEC counts, and RA activity, and (c) to correlate EPC and CEC counts with levels of serum markers of synovitis or endothelial injury.

## Materials and methods

### Patients

The study involved 59 RA patients (54 females; mean age of 54 ± 15 years) fulfilling the RA American College of Rheumatology criteria [21]. RA patients were consecutively enrolled during a 6-month period regardless of disease activity and underwent a routine clinical examination that included the calculation of 28-joint disease activity score (DAS-28). The patients' characteristics are summarized in Table 1. Ongoing biologic therapies included tumor necrosis factor (TNF) blockers (etanercept, adalimumab, or infliximab) in 11 patients and anti-CD20 (rituximab) in 12 patients. Thirty-six healthy volunteers (26 females; mean age of 53 ± 19 years) coming from our first study served as controls [20]. Exclusion criteria for all subjects were cardiovascular events and conventional cardiovascular risk factors (diabetes, hypertension, and past medical history of coronary artery disease and smoking) except for three RA patients with controlled systemic hypertension. None of the patients had been treated previously with statins, a drug known to be associated with increased EPC levels [22,23]. All patients and volunteers gave informed consent for all procedures, which were carried out with local ethics committee approval Comité de Protection des Personnes, Ile de France III (CPPP IDF III).

**Table 1:** Rheumatoid arthritis study population

Laboratory and clinical data	RA patients (n = 59)
Disease duration in days, mean ± SD	16 ± 11
Erosive RA, number (percentage)	54 (92)
Positive rheumatoid factor, >10 IU, ELISA, number (percentage)	52 (88)
Positive anti-CCP antibodies, >10 IU, ELISA, number (percentage)	52 (88)
ESR in mm/hour, mean ± SD; ESR >28, number (percentage)	28 ± 21; 35 (59)
CRP in mg/dL, mean ± SD; CRP >15, number (percentage)	25 ± 41; 23 (39)
DAS-28, mean ± SD	4.36 ± 1.7
DAS-28 < 2.6, number (percentage)	11 (19)
2.6 < DAS-28 < 5.1, number (percentage)	21 (35)
DAS-28 > 5.1, number (percentage)	27 (46)
Methotrexate, number (percentage)	49 (83)
Low dose of prednisone, ≤10 mg/day, number (percentage)	54 (92)
Anti-tumor necrosis factor agents, number (percentage)	11 (17)
Anti-CD20 agents, number (percentage)	12 (20)

anti-CCP, anti-cyclic citrullinated peptide; CRP, C-reactive protein; DAS-28, 28-joint disease activity score; ELISA, enzyme-linked immunosorbent assay; ESR, erythrocyte sedimentation rate; RA, rheumatoid arthritis; SD, standard deviation.

### Flow cytometry quantification

EPCs were quantified by fluorescence-activated cell sorting (FACS) as previously described [20]. Briefly, peripheral blood mononuclear cells (PBMCs) were first depleted of positive lineage mononuclear cells (CD2^+^, CD3^+^, CD14^+^, CD16^+^, CD19^+^, CD24^+^, CD56^+^, and CD66b^+ ^cells) by human progenitor cell enrichment cocktail (RosetteSep^®^; StemCell Technologies, Vancouver, BC, Canada), and secondly subjected to triple-labelling with anti-CD133-phycoerythrin (PE) (Miltenyi Biotec, Paris, France), anti-VEGFR-2 (KDR)-allophycocyanin (APC) (R&D Systems, Minneapolis, MN, USA), and anti-CD34 or anti-CD105-fluorescein isothiocyanate (FITC) (BD Biosciences, Le Pont de Claix, France) antibodies. A preincubation of an FcR-blocking reagent (Miltenyi Biotec) was performed to inhibit non-specific binding, and identical IgG isotypes served as negative controls. Third, viable PBMCs were discriminated by 7-aminoactinomycin D (7AAD) labelling. The EPC and CEC populations were finally identified as Lin^-^/7AAD^-^/CD34^+^/CD133^+^/VEGFR-2^+ ^cells and Lin^-^/7AAD^-^/CD105^+^/CD133^-^/VEGFR-2^+ ^cells, respectively. At least 500,000 events were analyzed, and results were expressed as the number of EPCs or CECs per million Lin^- ^mononuclear cells.

### Endothelial progenitor cell quantification by late-outgrowth colony-forming unit assay

In 53 RA patients and 35 controls with FACS quantification, we used a method of culture suitable for isolating late-outgrowth EPC-derived colonies [20]. The blood mononuclear cell fraction was collected by Ficoll (Pancoll, Dutcher, France) density gradient centrifugation and was resuspended in endothelial growth medium (EGM-2) (Lonza, Verviers, Belgium). Cells were then seeded on collagen-precoated 12-well plates (BD Biosciences) at 2 × 10^7 ^cells per well and stored at 37°C and 5% CO_2_. After 24 hours of culture, adherent cells were washed once with phosphate-buffered saline 1x and cultured in EGM-2 with daily changes until the quantification. Colonies of endothelial cells appeared between 9 and 26 days of culture and were identified as well-circumscribed monolayers of cells with a cobblestone appearance. EPC colonies were counted visually under an inverted microscope (Olympus, Paris, France).

### Enzyme-linked immunosorbent assays

In a random subgroup of 49 patients with RA and 10 healthy controls, levels of serum soluble vascular cell adhesion molecule-1 (sVCAM), stromal-derived factor-1 (SDF-1), human cartilage glycoprotein-39 (YKL-40), and cartilage oligomeric matrix protein (COMP) -- markers of endothelial injury, progenitor mobilization, synovitis, and synovitis, respectively -- were measured by enzyme-linked immunosorbent assay (R&D Systems; Kamiya Biomedical Company, Seattle, WA, USA; and Quidel Corporation, San Diego, CA, USA) in accordance with the manufacturers' instructions.

### Data analysis

All data are presented as median (range) unless otherwise stated. Comparisons were performed by non-parametric Mann-Whitney, Kruskal-Wallis, or Spearman rank correlation (*r*) tests, when appropriate. The chi-square test was used to compare categorical variables. *P *values are two-tailed, and *P *values of not more than 0.05 were considered statistically significant.

## Results

### Endothelial progenitor cell and circulating endothelial cell levels in rheumatoid arthritis

**Figure 1:**
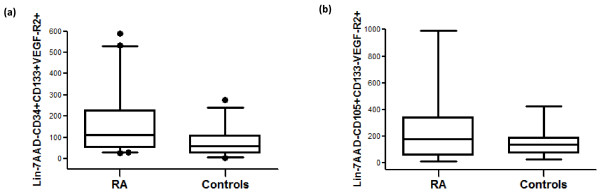
**Endothelial progenitor cell (EPC) and circulating endothelial cell (CEC) levels in rheumatoid arthritis (RA) patients compared with controls (cells per 10^6 ^lineage-negative [Lin^-^] mononuclear cells)**. **(a) **Higher EPC levels (Lin^-^7AAD^-^CD34^+^CD133^+^VEGFR-2^+^) in RA patients (n = 59) than in controls (n = 36) (*P *< 0.001). **(b) **No significant difference between CEC levels (Lin^-^7AAD^-^CD105^+^CD133^-^VEGFR-2^+^) in RA patients (n = 59) and controls (n = 15). 7AAD, 7-aminoactinomycin D; VEGFR-2, vascular endothelial growth factor receptor-2.

**Figure 2:**
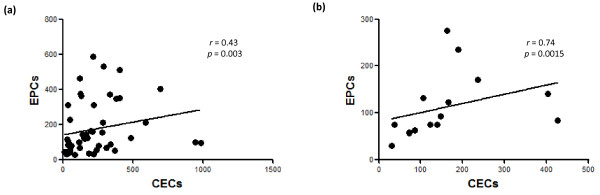
**Positive correlation between endothelial progenitor cell (EPC) counts and circulating endothelial cell (CEC) counts (cells per 10^6 ^lineage-negative mononuclear cells)**. **(a) **Patients with rheumatoid arthritis. **(b) **Controls. Correlation coefficient *r *and *P *values are indicated.

EPC level (Lin^-^/7AAD^-^/CD34^+^/CD133^+^/VEGFR-2^+^) was significantly higher in RA patients than in controls (112 [27 to 588] versus 60 [5 to 275] EPCs; *P *= 0.0007) (Table 2 and Figure 1a). The two Lin^-^/7AAD^-^/CD133^+^/VEGFR-2^+ ^and Lin^-^/7AAD^-^/CD34^+^/VEGFR-2^+ ^subpopulations were also significantly higher in RA patients (Table 2). The CEC population (Lin^-^/7AAD^-^/CD105^+^/CD133^-^/VEGFR-2^+ ^cells) was increased in RA patients compared with controls, although this did not reach statistical significance (Table 2 and Figure 1b). The CEC and EPC levels in RA patients as well as in controls were correlated (*r *= 0.43 and 0.74, *P *= 0.003 and 0.0015, respectively) (Figure 2).

**Table 2:** Absolute number of stem cells and circulating endothelial progenitor cells in peripheral blood (cells per 10^6 ^lineage-negative mononuclear cells)

Cells per 10^6 ^Lin^- ^mononuclear cells	RA patients (n = 59)	Controls (n = 36)	*P *value
Lin^-^/7AAD^-^/CD34^+^/CD133^+^			0.0002
Median (range)	2,484 (213-26,613)	1,068 (123-2,270)	
25%-75% percentile	1,141-5,943	610.5-1,575	
Lin^-^/7AAD^-^/CD34^+^/VEGFR-2^+^			0.0026
Median (range)	259 (27-588)	103 (11-1,105)	
25%-75% percentile	124-493	49.75-193.8	
Lin^-^/7AAD^-^/CD133^+^/VEGFR-2^+^			0.0276
Median (range)	151 (45-964)	125 (25-555)	
25%-75% percentile	93-387	99.25-192.8	
Lin^-^/7AAD^-^/CD34^+^/CD133^+^/VEGFR-2^+ ^(EPCs)			0.0007
Median (range)	112 (27-588)	60 (5-275)	
25%-75% percentile	53-227	26-109.8	
Lin^-^/7AAD^-^/CD105^+^/CD133^-^/VEGFR-2^+^(CECs)			0.1727
Median (range)	173 (12-989)	139 (30-426)^a^	
25%-75% percentile	57-341	73-189	
Ratio EPCs/CECs, median (range)	1 (0.07-4.28)	1 (0.2-2.02)^a^	0.3946

^a^Circulating endothelial cell (CEC) number has been evaluated in 15 healthy controls. 7AAD, 7-aminoactinomycin D; DAS-28, 28-joint disease activity score; EPC, endothelial progenitor cell; Lin^-^, lineage-negative; RA, rheumatoid arthritis; VEGFR-2, vascular endothelial growth factor receptor-2.

### Association between endothelial progenitor cell levels and disease activity

**Figure 3:**
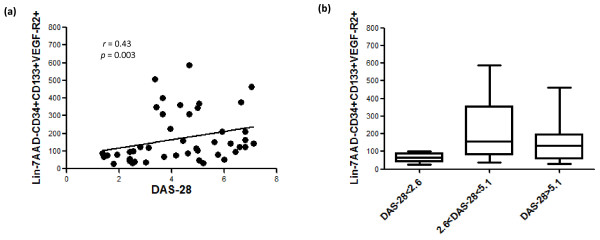
**Associations of endothelial progenitor cell (EPC) levels in rheumatoid arthritis (RA) with disease activity**. Correlation between EPC counts and disease activity **(a) **and association of lower EPC levels in different groups of RA patients according to disease activity score (*P *< 0.01) **(b)**. 7AAD, 7-aminoactinomycin D; DAS-28, 28-joint disease activity score; Lin^-^, lineage-negative; VEGFR-2, vascular endothelial growth factor receptor-2.

In RA patients, EPC levels correlated with DAS-28 (*r *= 0.43, *P *= 0.003, Spearman test) (Figure 3a). In addition, EPC levels were significantly decreased in patients fulfilling DAS-28 remission criteria when compared with moderate (2.6 < DAS-28 < 5.1) or high (DAS-28 >5.1) activities (*P *= 0.007, Kruskal-Wallis test) (Figure 3b). No association was found between EPC levels and high values of erythrocyte sedimentation rate (ESR) (>28) (*P *= 0.9) or between EPC levels and high values of C-reactive protein (>15) (*P *= 0.7). Swollen joint counts did not correlate with EPC values (*r *= 0.23, *P *= 0.99). There was no association between EPC number and age, disease duration, and other disease features, including treatments (low doses of corticosteroids and methotrexate). In regard to biologic therapy, patients receiving TNF blockers or anti-CD20 did not have different EPC levels (median [range] 88 [27 to 529], *P *= 0.9922 and 83.5 [33 to 345], *P *= 0.1906, respectively). Likewise, CEC levels did not correlate with RA characteristics or treatments.

### Number of late-outgrowth endothelial progenitor cell colony-forming units in rheumatoid arthritis

**Figure 4:**
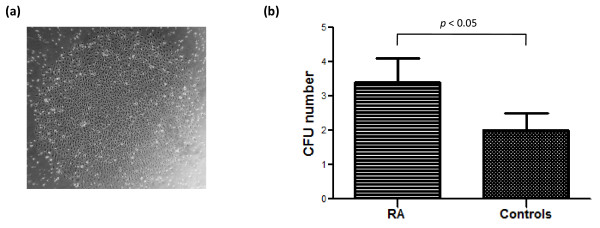
**Late-outgrowth endothelial progenitor cell colony-forming unit (CFU) number in rheumatoid arthritis (RA) patients and controls**. **(a) **Representative photomicrograph of late-outgrowth CFUs in culture after 15 days from one RA patient (×40 magnification). **(b) **Increase of CFU number in RA patients (*P *< 0.05).

Endothelial colony formation has previously been used as an alternative method to detect endothelial progenitors in PBMCs [20]. CFU assays were performed in association with FACS quantification in 53 RA patients and 35 controls. EPC-CFUs appeared at the ninth day of PBMC culture at the earliest and were confirmed by a typical morphology of a well-delineated colony of cells with a cobblestone appearance (Figure 4a). The percentage of RA patients displaying EPC colony formation was significantly higher than that of controls (74% versus 57%; *P *= 0.029) in accordance with their higher EPC levels (118.5 [29 to 588] versus 84 [19 to 275]; *P *= 0.049). Furthermore, comparison of the mean number of EPC-CFUs measured in RA patients and controls revealed an increase in CFU number in RA patients (3.4 ± 0.7 versus 2 ± 0.5; *P *= 0.048) (Figure 4b). In the RA population, CFU numbers correlated with none of RA characteristics or treatments.

### Lack of association between serum markers and endothelial progenitor cell levels

Different serum markers -- including sVCAM (677 [294 to 2,624] versus 512 [371 to 738] ng/mL; *P *= 0.018), YKL-40 (88 [24 to 256] versus 50 [15 to 59] ng/mL; *P *= 0.0029), and COMP (2.4 [1.2 to 4.4] versus 1.3 [0.8 to 1.5] μg/mL; *P *< 0.0001) -- were found to be significantly increased in RA patients as compared with healthy controls. There was no significant difference for SDF-1 concentration (3,690 [73 to 6,973] versus 3,562 [1,540 to 6,451] pg/mL). However, values of EPCs in RA patients were unrelated to any of the above serum markers. Also, CEC levels were not linked with serum markers of synovitis but were significantly higher in RA patients with a high sVCAM level (>1,000 ng/mL; *P *= 0.0035).

## Discussion

Our results obtained by using a well-characterized definition of late-outgrowth EPCs in a relatively large number of patients show enhanced levels of this cell population and relationships with RA disease activity. Available data have reported conflicting results about the EPC counts in this inflammatory condition. Several methodological issues could explain such discrepancies. We herein followed recent recommendations and used a previously validated method for late-outgrowth EPC enumeration [20].

The quantification of EPCs by flow cytometry first requires enrichment techniques to select a correct number of this scarce population and a specific marker combination to select the subpopulation of hemangioblastic EPCs. In culture, these 'true' angioblast-like EPCs are represented by cells that enable late outgrowth with higher proliferative potential, while endothelial cell colonies that appear early might more preferentially originate from monocytes or CECs [11,24]. In the study herein, we excluded the monocytic EPC subpopulation from the quantification and thus selected hemangioblastic EPCs by the lineage-positive cell depletion including CD14^+ ^cells. We also extended the circulating EPC definition with an additional marker of viability to select non-apoptotic cells. One may suggest that the several steps required by our technique may induce procedural loss of progenitors which are prone to undergo apoptosis. However, the controlled design of our study limits this potential bias but this will need additional work. In parallel, EPC counts were also determined by the selection in culture of the late-outgrowth EPCs according to the delays before their appearance.

None of the previous studies that reported EPC levels in RA patients was based on these methods. The previous data quantified, conversely to our study, circulating EPCs in whole blood with only three surface markers (CD34/CD133/VEGFR-2) [8-10,16]. These authors also assessed EPC-CFU numbers, focusing on the 'early outgrowth' subpopulation and finding or not finding results consistent with those of flow cytometry quantification. These methodological differences may account for the discrepancy with regard to our results. Indeed, differences between the various EPC studies may not relate to the characteristics of the RA population enrolled. Our population of RA patients, issued from consecutive inclusions, did not display differences with other studies based on criteria known to modulate EPC levels -- such as age (mean of 53 to 59 years) and frequency of use of methotrexate or low doses of corticosteroids -- or on the choice to exclude patients with previous cardiovascular events [25-27]. In addition, it is noteworthy that EPC counts did not differ according to the use of biologics, although the cross-sectional design limits the analysis of the influence of such therapies in our RA patients. The only specificity of our RA patients may be the relatively long disease duration in comparison with other works. Nevertheless, disease duration was never reported to be associated with EPC levels and thus may not account for our findings. We excluded RA patients with cardiovascular risk factors in order to rule out the bias of the specific effects of atheroma on EPC counts and thus to focus on relationships between disease activity and EPC counts as this has been done in many previous studies [9,10]. One may suggest that this may have introduced a selection bias and the use of this exclusion criterion may have obscured a negative influence of cardiovascular risk factors on EPC counts.

We herein provide the demonstration of the identification of the late-outgrowth subset by the association between circulating cell counts and culture isolations. Indeed, two different subpopulations of EPCs, namely early and late EPCs, can be derived from peripheral blood depending on the different culture methods and times [24,28]. Although both EPCs express endothelial markers, they have different morphologies, patterns of growth, and angiogenic properties and thus might have different roles in neovasculogenesis [24,29-31]. Late-outgrowth EPCs that represent the progenitors with the more genuine endothelial properties such as tube-forming activity *in vitro *and *in vivo *need to be better characterized in the context of inflammatory conditions. Previously, the late EPC subpopulation has been studied in systemic sclerosis by our group and their endothelial properties confirmed by angiogenic tests [32]. As reported in systemic sclerosis, we observed that the RA patients, having high Lin^-^7AAD^-^CD34^+^CD133^+^VEGFR-2^+^, displayed a higher number of EPC-CFUs. However, the size of the sample reduced by the non-systematic achievement of EPC-CFUs could explain the limited increase of EPC-CFU numbers in RA patients as well as the lack of association with disease activity and will need larger studies.

We thus assume that our EPC definition allows a well-characterized quantification of late-outgrowth EPCs. In RA, our data support the contribution of late-outgrowth EPCs to synovitis according to our finding of a strong link with disease activity. The direct correlation of EPC counts with DAS-28 levels is strengthened by the fact that RA patients in remission displayed EPC levels comparable to those of controls. Together with evidence of CD133/VEGFR-2^+ ^cells in RA synovial tissue [17], our results emphasize a key role for vasculogenesis and EPC mobilization in RA.

Preliminary data on CEC outcome in vascular diseases have suggested a relationship between the detachment of mature CECs and vascular hurting [33]. We concomitantly determined the value of CECs as compared with EPCs. We found a correlation in RA between these two circulating cell levels but CEC levels in RA did not differ for the ones in controls. Using a CD146 immunoselection in whole blood, one study found enhanced CEC levels but failed to identify a specific association with blood inflammatory markers [34]. The best combination of surface markers including exclusion of dead cells by viability marker seems to be required for CEC quantification.

One of the pitfalls of EPC quantification in RA is the potential involvement of atherosclerosis in EPC changes [35,36]. However, we excluded from our study individuals with classical cardiovascular risk factors and also those with previous clinical events. Furthermore, we did not find an increase of CEC levels in our RA population which reflects endothelial injury. Indeed, we presume that EPC increase relates to RA disease activity and synovial inflammation, although measurement of infra-clinical atheroma would be necessary to definitely rule out endothelial dysfunction.

We then attempted to correlate EPC counts with blood markers reflecting inflammation (ESR), endothelium injury (sVCAM), or synovial involvement (COMP and YKL-40). While we failed in the identification of any link, sVCAM, COMP, and YKL-40 were found to be increased in RA patients, thus confirming the activity of the disease in our sample of RA patients. We assume that, despite the lack of a link with serum markers, the identification of a relationship between EPCs and DAS-28 is highly relevant. EPC count is probably influenced by several factors, including inflammation, vascular injury, and potentially the immune response with bone marrow changes. The multifactorial regulation probably precludes the identification of a correlation with one single serum marker. Therefore, EPCs could represent a new 'integrative' biomarker. Its predictive value on disease outcome, including both articular and cardiovascular issues, will have to be evaluated in upcoming prospective studies.

## Conclusions

We demonstrate enhanced levels of late-outgrowth EPCs in RA and a relationship with disease activity. This supports the implication of vasculogenesis in the perpetuation of the synovitis that occurs in RA. Late-outgrowth EPC isolation also offers a unique opportunity to determine an RA endothelial signature.

## Abbreviations

7AAD: 7-aminoactinomycin D; CEC: circulating endothelial cell; CFU: colony-forming unit; COMP: cartilage oligomeric matrix protein; DAS-28: 28-joint disease activity score; EGM-2: endothelial growth medium; EPC: endothelial progenitor cell; ESR: erythrocyte sedimentation rate; FACS: fluorescence-activated cell sorting; KDR: kinase-insert domain receptor; Lin: lineage; PBMC: peripheral blood mononuclear cell; RA: rheumatoid arthritis; SDF-1: stromal-derived factor-1; sVCAM: soluble vascular cell adhesion molecule-1; TNF: tumor necrosis factor; VEGFR-2: vascular endothelial growth factor receptor-2; YKL-40: human cartilage glycoprotein-39.

## Competing interests

The authors declare that they have no competing interests.

## Authors' contributions

VJ contributed to the study design and did most of the experimental procedures and data analysis. JA, AP, and BR participated in some of the experimental procedures and the data analysis. YA recruited the patients, analyzed the results, and supervised the study. AK, CB, and GU contributed to the revision of the manuscript. All authors read and approved the final manuscript.
